# In non-transformed cells Bak activates upon loss of anti-apoptotic Bcl-X_L_ and Mcl-1 but in the absence of active BH3-only proteins

**DOI:** 10.1038/cddis.2015.341

**Published:** 2015-11-26

**Authors:** D Senft, A Weber, F Saathoff, C Berking, M V Heppt, C Kammerbauer, S Rothenfusser, S Kellner, Z Kurgyis, R Besch, G Häcker

**Affiliations:** 1 Department of Dermatology and Allergology, Ludwig-Maximilian University, 80337 Munich, Germany; 2 Institute of Medical Microbiology and Hygiene, University Medical Center Freiburg, 79104 Freiburg, Germany; 3 Division of Clinical Pharmacology, Department of Internal Medicine, Ludwig-Maximilian University, 80336 Munich, Germany; 4 Center of Integrated Protein Science CIPS-M, Division of Clinical Pharmacology, Department of Internal Medicine, Ludwig-Maximilian University, 80336 Munich, Germany; 5 Section of Gastroenterology and Endocrinology, Medizinische Klinik Innenstadt, Ludwig-Maximilian University, 80336 Munich, Germany; 6 Department of Dermatology and Allergology, University of Szeged, 6720 Szeged, Hungary

## Abstract

Mitochondrial apoptosis is controlled by proteins of the B-cell lymphoma 2 (Bcl-2) family. Pro-apoptotic members of this family, known as BH3-only proteins, initiate activation of the effectors Bcl-2-associated X protein (Bax) and Bcl-2 homologous antagonist/killer (Bak), which is counteracted by anti-apoptotic family members. How the interactions of Bcl-2 proteins regulate cell death is still not entirely clear. Here, we show that in the absence of extrinsic apoptotic stimuli Bak activates without detectable contribution from BH3-only proteins, and cell survival depends on anti-apoptotic Bcl-2 molecules. All anti-apoptotic Bcl-2 proteins were targeted via RNA interference alone or in combinations of two in primary human fibroblasts. Simultaneous targeting of B-cell lymphoma-extra large and myeloid cell leukemia sequence 1 led to apoptosis in several cell types. Apoptosis depended on Bak whereas Bax was dispensable. Activator BH3-only proteins were not required for apoptosis induction as apoptosis was unaltered in the absence of all BH3-only proteins known to activate Bax or Bak directly, Bcl-2-interacting mediator of cell death, BH3-interacting domain death agonist and p53-upregulated modulator of apoptosis. These findings argue for auto-activation of Bak in the absence of anti-apoptotic Bcl-2 proteins and provide evidence of profound differences in the activation of Bax and Bak.

The regulated elimination of cells by apoptosis is a key mechanism of development, tissue homeostasis and defense. In vertebrates, apoptosis is regulated through two pathways, the death receptor-mediated (extrinsic) and the mitochondrial (intrinsic) pathway, which is activated by numerous apoptotic stimuli. Mitochondrial apoptosis is characterized by loss of mitochondrial outer membrane integrity and the release of mitochondrial intermembrane space proteins, most notably cytochrome *c*, which leads to the activation of the caspase-9 and effector caspases.^1^

Release of cytochrome *c* is governed by proteins of the B-cell lymphoma 2 (Bcl-2) family.^2^ The Bcl-2 family consists of three groups, whose expression and interaction decide cell survival. The anti-apoptotic Bcl-2 proteins include Bcl-2, Bcl-X_L_ (B-cell lymphoma-extra large), Bcl-w (Bcl-2-like protein 2), Mcl-1 (myeloid cell leukemia sequence 1) and A1 (Bcl-2-related protein A1). The pro-apoptotic group of BH3-only proteins (containing a BH3-domain: Bim (Bcl-2-interacting mediator of cell death), Bid (BH3-interacting domain death agonist), Puma (p53-upregulated modulator of apoptosis), Noxa (Phorbol-12-myristate-13-acetate-induced protein 1), Bad (Bcl-2-associated death promoter), Bik (Bcl-2-interacting killer) and Hrk (activator of apoptosis hara-kiri)) activate the pro-apoptotic effectors Bcl-2-associated X protein (Bax) and Bcl-2 homologous antagonist/killer (Bak). Bax and Bak can replace each other in most situations, but the presence of one of them is required for mitochondrial apoptosis. Upon activation Bax and Bak form oligomers in the outer mitochondrial membrane and cause the release of cytochrome *c*. How Bax and Bak are activated is still under debate. Different activation models have been proposed and investigated.

According to the direct activation model BH3-only proteins can directly, by physical interaction activate Bax and Bak.^3^ The model was derived in studies investigating synthetic BH3-domain peptides in *in vitro* systems, that is, isolated mitochondria or liposomes, where peptides encompassing the BH3-domains of Bim or Bid (‘activator' BH3-only proteins) were able to activate Bax. Peptides derived from the BH3-only proteins Bad, Bik, Hrk, Noxa or Puma did not activate Bax directly. However, these peptides can bind to anti-apoptotic Bcl-2 proteins with varying preferences.^4^ As this may neutralize a combination of anti-apoptotic proteins it may facilitate Bax/Bak activation by activator BH3-only proteins. Consequently, this group of BH3-only proteins has been named ‘sensitizer' or ‘derepressor' BH3-only proteins.^3, 5, 6, 7^ The direct activation model has received recent support by structural studies of activator BH3-domains bound to Bax.^8^ That study also found that the BH3-only peptides used previously lacked a residue that is important in the activation of Bax, and the previous results may have to be reconsidered. Indeed, a recent study illustrates that placing the BH3-domain from the various BH3-only proteins into intact Bid protein enhances Bax/Bak-activating capacity of the BH3-domains of Bid, Bim, Puma, Bmf (Bcl-2-modifying factor), Bik and Hrk.^9^

The displacement (or indirect activation) model on the other hand posits that Bax and Bak are held in check by anti-apoptotic Bcl-2 proteins and auto-activate when this interaction is broken by BH3-only proteins (displacement). BH3-only proteins can bind to anti-apoptotic Bcl-2 proteins and upon apoptotic stimulation may cause the displacement of these proteins from Bax and Bak, which may lead to the activation of effectors. BH3-peptides derived from Bim and Puma can bind to all anti-apoptotic Bcl-2 proteins and its corresponding proteins exert killing upon overexpression, whereas Bad, Bmf, Bid, Bik, Hrk and Noxa display binding patterns restricted to certain anti-apoptotic Bcl-2 proteins.^4^ It was therefore suggested that Bax/Bak activation requires the neutralization/displacement of several anti-apoptotic proteins, which may be achieved by one BH3-only protein with broadly binding characteristics (such as Bim) or by the combination of BH3-only proteins with restricted binding capabilities (for instance Bad plus Noxa).^10, 11^

The models have been further refined; the ‘embedded together' model additionally considers the dynamic interaction of the proteins with the mitochondrial membrane,^12^ and it has been proposed that the models can be unified by taking two ‘modes' of inhibition into account: anti-apoptotic Bcl-2 proteins have a dual function in inactivating both, BH3-only proteins and effectors. Pro-apoptotic signals cause the release of activator BH3-only proteins from sequestration with anti-apoptotic Bcl-2 proteins. Free BH3-only proteins directly activate effectors, however, cell death may still not be initiated because the effectors are then held in check by anti-apoptotic Bcl-2 proteins. Free activator BH3-only proteins are required to activate effectors.^13^

This model unifies the two above models in the sense that it incorporates aspects of both, inhibition and displacement as well as direct activation. However, the core difference between the (direct) activation and the displacement model appears to be irreconcilable: in the activation model Bax and Bak are inactive unless receiving a stimulus from BH3-only proteins whereas in the displacement model they are active unless bound to anti-apoptotic proteins. Thus, in the absence of all other proteins one model predicts that Bax/Bak are active, the other that they are inactive. Obviously they cannot be both.

The direct activation model has initially been established with Bax and the displacement model with Bak. The data are very strong that Bax is activated by direct interaction with BH3-only proteins. Recombinant Bak can also be directly activated by recombinant tBid,^14^ and Bid/BH3-chimaeras can activate recombinant Bak missing its C terminus.^9^ However, since Bak is normally inserted into the outer mitochondrial membrane where it may be bound to numerous other Bcl-2-family members, it has been difficult directly to test activation of Bak in the physiological situation.

One possibility to ‘unify' the original models may be in a model where Bax is physiologically activated by direct activation (Bax is inactive until receiving a signal through BH3-only proteins) whereas Bak is activated indirectly (auto-activates when the inhibition by Bcl-2-like proteins is relieved). Here we test this possibility of indirect Bak activation. We targeted anti-apoptotic Bcl-2 family proteins using RNAi. In this setting, protein concentrations and conditions are physiological, which avoids some of the problems associated with overexpression or cell-free experiments. Non-malignant cells may respond differently to the loss of anti-apoptotic Bcl-2 proteins compared with tumor cells.^15^ In this study, using non-malignant cells, we targeted all anti-apoptotic Bcl-2 molecules in combinations of two. In the absence of apoptotic stimuli we observed that the combined loss of Bcl-X_L_ and Mcl-1 was sufficient to induce apoptosis. The direct activator proteins Bid, Bim and Puma were not needed. These observations provide evidence for indirect activation of Bak.

## Results

### Combined targeting of Bcl-X_L_ and Mcl-1 induces apoptosis in primary human cells

**Figure 1:**
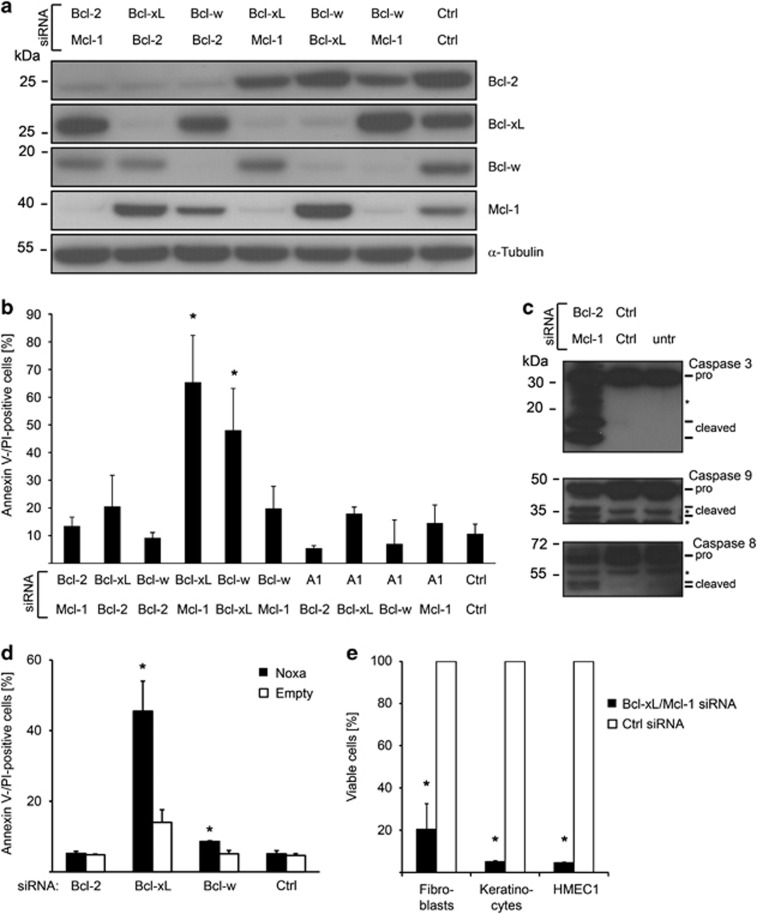
Combined inhibition of Bcl-X_L_ and Mcl-1 induces apoptosis in primary cells. (**a**) Primary human fibroblasts were simultaneously transfected with two siRNAs as indicated. Protein expression was measured 48 h after transfection by immunoblotting. *α*-Tubulin served as loading control. Results are representative of three independent experiments. (**b**) Cell death of fibroblasts was measured by quantifying Annexin V- and propidium iodide (PI)-positive cells 3 days after siRNAs treatment. Data show means±S.D. of three independent experiments. (**c**) Caspase activation in fibroblasts assessed by immunoblotting 48 h after siRNA treatment. Data are representative of two independent experiments. (**d**) Fibroblasts were transfected with indicated siRNAs. Forty-eight hour after siRNA transfection cells were transfected with a Noxa-encoding or empty plasmid. Cell death was measured 24 h after plasmid transfection. Data show means±S.D. of three independent experiments. (**e**) Cell viability was assessed 3 days after siRNA transfection in primary human fibroblasts, primary human keratinocytes, and immortalized human microvascular endothelial cells (HMEC1). Data are given as means±S.D. of three independent transfections. *P≤0.05

We recently investigated anti-apoptotic Bcl-2 proteins to evaluate a strategy to induce apoptosis in a tumor-specific way and observed that – in contrast to tumor cells – targeting of a single anti-apoptotic Bcl-2 protein by RNAi does not affect survival of primary (non-transformed) human fibroblasts.^15^ To understand whether any anti-apoptotic proteins are required for survival we analysed the effect of combined simultaneous RNAi to two anti-apoptotic Bcl-2 proteins. All combinations of two of the anti-apoptotic Bcl-2 proteins, Bcl-2, Bcl-X_L_, Bcl-w, Mcl-1 and A1 were tested and efficient targeting of all proteins was achieved (Figure 1a and ref. 15 for A1). Although most combinations did not lead to significant cell death, targeting of Bcl-X_L_ and Mcl-1 killed ~60% of cells within 3 days (Figure 1b; a less complete study in HeLa cells has also reported that simultaneous targeting of Bcl-X_L_ and Mcl-1 induces apoptosis^16^). To a lesser extent, targeting of Bcl-X_L_ and Bcl-w caused cell death, however, induction of Noxa was observed there (data not shown). Noxa inactivates Mcl-1, and experimental expression of Noxa sensitized the cells to RNAi against Bcl-X_L_ alone (see below). Cell death upon targeting of Bcl-X_L_/Bcl-w may therefore not be due to the loss of Bcl-2-function but the Noxa-mediated inactivation of Mcl-1, together with RNAi against Bcl-X_L_; this targeting may thus have the same molecular effect as RNAi against the combination of Bcl-X_L_ and Mcl-1. Therefore, the relevance of Bcl-X_L_/Bcl-w for cell death cannot be addressed in this approach. Activation of caspase-9 and caspase-3 was observed by western blotting (Figure 1c). Cleavage of caspase-8 was also observed, which very likely was subsequent to the activation of effector caspases.

To confirm this finding in another setting, we ectopically expressed Noxa along with siRNA-mediated targeting of Bcl-2, Bcl-X_L_ and Bcl-w. Only upon targeting of Bcl-X_L_, Noxa expression led to apoptosis. As Noxa specifically targets Mcl-1 this is additional evidence that specifically Mcl-1 inactivation along with Bcl-X_L_ targeting leads to cell death (Figure 1d). Finally, we tested this combination in different cell types and compared primary human fibroblasts (mesenchymal cells) with primary human keratinocytes (epithelial cells) and immortalized human microvascular endothelial cells. Targeting of Bcl-X_L_ and Mcl-1 caused cell death also in these cell types (Figure 1e). These data show that specifically the combined loss of Bcl-X_L_ and Mcl-1 induces cell death in a number of different primary cells without further stimulus.

### Apoptosis upon targeting of Bcl-X_L_ and Mcl-1 is mediated by Bak

**Figure 2:**
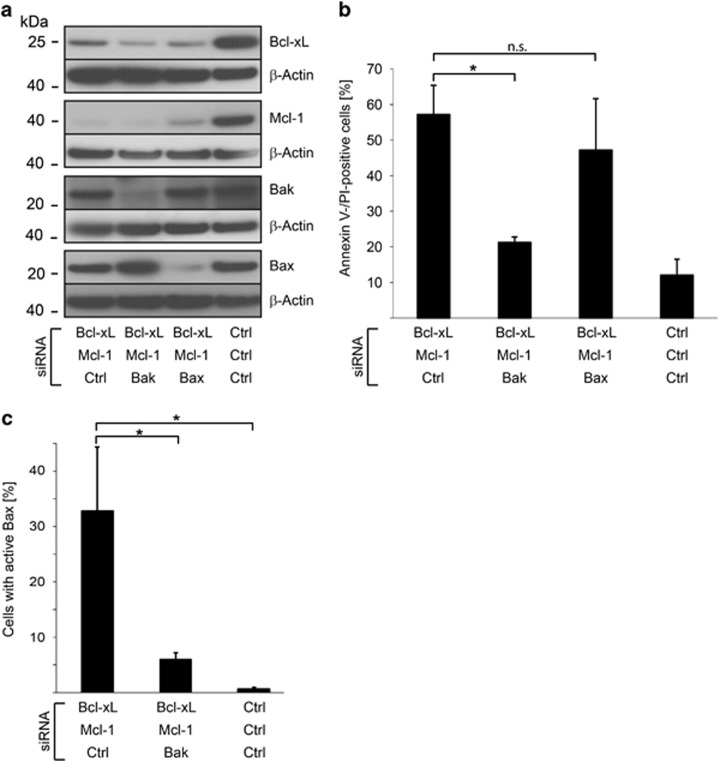
Apoptosis upon inhibition of Bcl-X_L_ and Mcl-1 is mediated by Bak. (**a**) Primary human fibroblasts were transfected with indicated siRNAs. Protein expression was measured 48 h after siRNA treatment. Results are representative of three independent experiments. (**b**) Fibroblasts were treated as described in **a** and cell death was quantified 3 days after transfection. Data are given as means±S.D. of three independent experiments. (**c**) Fibroblasts were treated as described in **a** and activation of Bax was quantified by cytometry after staining with an antibody specific for activated Bax. Data show means±S.D. of at least three independent experiments. *P≤0.05; n.s., not significant

We next tested the relevance of the effector Bcl-2 proteins Bax and Bak for apoptosis induced by the loss of Bcl-X_L_ and Mcl-1. We were successful in knocking-down expression of either effector together with Bcl-X_L_/Mcl-1 (Figure 2a). Surprisingly, targeting of Bak almost completely blocked cell death, whereas targeting of Bax had no significant effect on cell death (Figure 2b). We also measured the proportion of cells with activated Bax, using staining for the 6A7 epitope in active Bax. Bax was found to be activated upon targeting of Bcl-X_L_/Mcl-1 (Figure 2c). However, no activation was seen when Bak had been targeted at the same time, suggesting that Bax activation is secondary to Bak activation but Bax cannot be activated in its absence (Figure 2c).

### Apoptosis upon targeting of Bcl-X_L_ and Mcl-1 in murine embryonic fibroblasts depends on Bak

**Figure 3:**
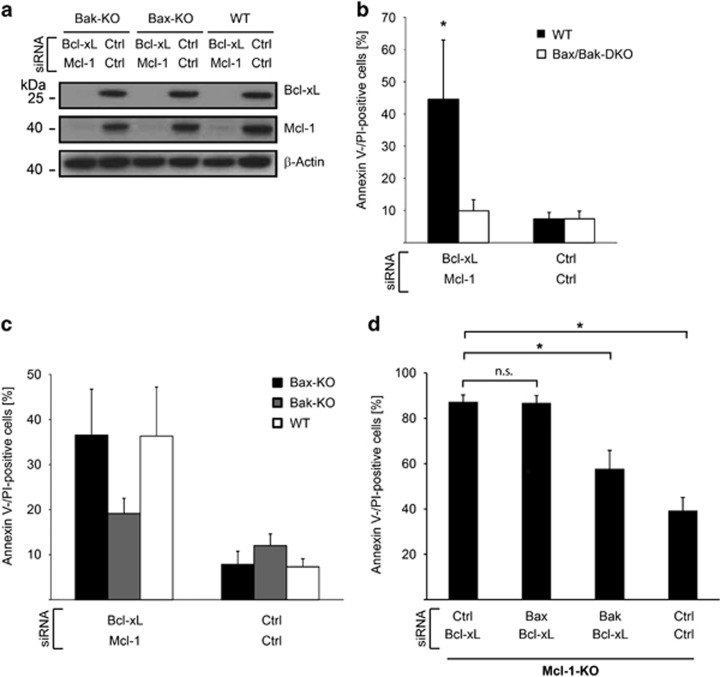
Bak-dependent apoptosis upon inhibition of Bcl-X_L_ and Mcl-1 in murine embryonic fibroblasts from knockout mice. (**a**) MEFs from Bax- or Bak-deficient, or WT mice were treated with siRNAs for 3 days and protein expression was measured. Results are representative of three independent experiments. (**b**) MEFs from Bax/Bak-double-deficient or WT mice were treated with siRNAs and cell death was analyzed 3 days after transfection. Data show means±S.D. of three independent experiments. (**c**) MEFs from Bax- or Bak-deficient or WT mice were treated and analyzed as described in **b**. The small difference in cell death upon targeting Bcl-X_L_ and Mcl-1 in Bak-deficient cells was statistically significant (*P*=0.048, control *versus* Bak-deficient cells). (**d**) MEFs from Mcl-1-deficient mice were treated with indicated siRNAs and analyzed 24 h after treatment. Bcl-X_L_ inhibition is sufficient in these cells to induce cell death. Data show means±S.D. of three independent experiments. *P≤0.05; n.s., not significant

To confirm Bak dependency of apoptosis induced by the loss of Bcl-X_L_/Mcl-1, we used murine embryonic fibroblasts (MEFs) WT or deficient for Bak, Bax or double deficient for both. Knock-down of Mcl-1 and Bcl- X_L_ was also efficient in these cells (Figure 3a). Similar to human fibroblasts, targeting of Bcl-X_L_/Mcl-1-induced apoptosis in WT-MEFs, which was not observed in Bax/Bak-DKO-MEFs (Figure 3b). Whereas apoptosis in Bax-deficient MEFs was similar to the levels in WT-MEFs, it was substantially reduced in Bak-deficient MEFs (Figure 3c). There was a very small pro-apoptotic effect also in Bak-deficient cells (Figure 3C). This may be a BH3-only protein-dependent effect (for instance, the targeting of the anti-apoptotic proteins may release some Bim), or it may be that in the absence of Bcl-X_L_, which can retro-translocate Bax from mitochondria to the cytosol,^17^ the accumulation of Bax at mitochondria will have a small pro-apoptotic effect. The effect was however minuscule compared to the effect of Bak (suggested by the effect in Bax- *versus* Bak-deficient cell, Figure 3c).

We also tested Mcl-1-deficient MEFs. Here, targeting of Bcl-X_L_ was sufficient to induce cell death and again, cell death was dependent on Bak, but not on Bax (Figure 3d). Together, these results confirm the findings in murine embryonic fibroblasts in different settings of Bcl-2 protein family deficiency.

### Apoptosis upon Bcl-X_L_/Mcl-1 inhibition is initiated in the absence of altered BH3-only protein expression levels

**Figure 4:**
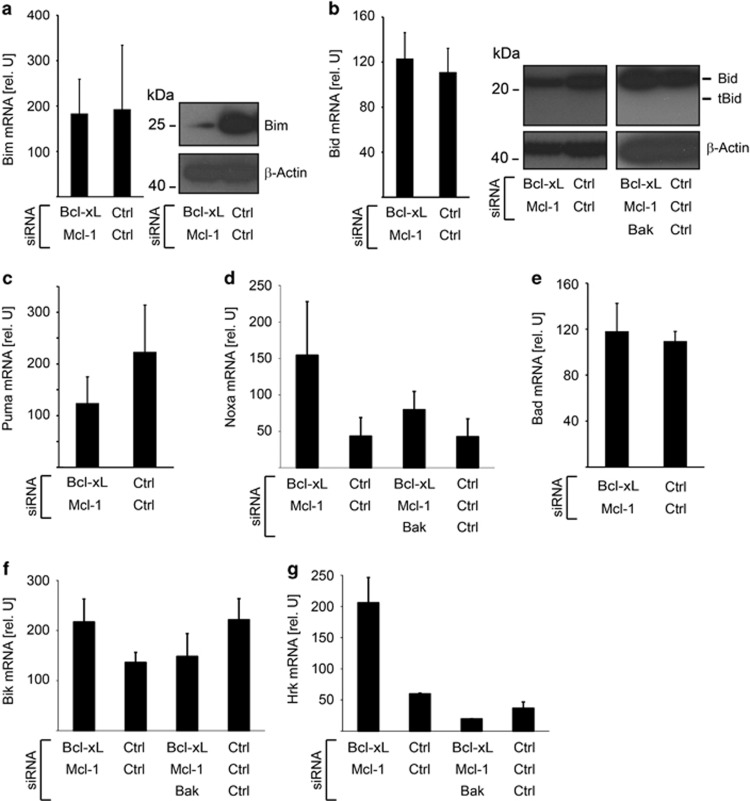
Apoptosis upon Bcl-X_L_/Mcl-1 inhibition is initiated in the absence of altered BH3-only protein expression levels. (**a**) Primary human fibroblasts were treated with Bcl-X_L_/Mcl-1 or Ctrl siRNAs. Expression of pro-apoptotic Bim was quantified 3 days after transfection on RNA (left panel) or protein level (right panel). Data show means±S.D. and immunoblots are representative of three independent transfections. (**b**) Analysis of pro-apoptotic Bid in fibroblasts treated with indicated siRNAs for 3 days. Data show means±S.D. and immunoblots are representative of three independent transfections. (**c**–**g**) Analysis of Puma, Noxa, Bad, Bik and Hrk in fibroblasts as described in **b**. Data show means±S.D. and immunoblots are representative of three independent transfections

Loss of Bcl-X_L_/Mcl-1 thus leads to the activation of Bak. This could be due either to the liberation of Bak from these two anti-apoptotic molecules or to the direct activation of Bak by BH3-only proteins, which may either be released from Bcl-X_L_/Mcl-1 or increased in expression. First, we tested expression levels of BH3-only proteins with known capacity to directly activate effector Bcl-2 proteins, namely Bim, Bid and Puma. Bim-mRNA was not altered upon targeting of Bcl-X_L_/Mcl-1 whereas protein levels were surprisingly reduced (Figure 4a). Bid mRNA was not altered; however, a slight reduction of Bid protein levels was observed, whereas tBid was not detectable (Figure 4b). It cannot fully be excluded that reduced Bid levels represent Bid activation even in the absence of detectable tBid. We therefore tested whether Bid levels were also reduced in the absence of Bak. Bid levels were not found to be reduced upon targeting of Bak (Figure 4b, right panel), suggesting that – if reduced Bid levels represent Bid activation – Bid may be activated downstream of Bak in an indirect manner but is not critical for apoptosis induction. Basal Puma mRNA levels were not significantly altered (Figure 4c) and Puma protein was not detectable in these cells (data not shown). Levels of Noxa, often regulated via transcription, were assessed by RT-PCR. Noxa mRNA levels were increased, but in a Bak-dependent manner suggesting an activation downstream of Bak (Figure 4d). Noxa specifically antagonizes Mcl-1 and A1.^4^ Because Mcl-1 is already targeted by siRNA and combined Bcl-X_L_/A1 inhibition did not lead to apoptosis (Figure 1b). Noxa is unlikely to affect apoptosis upon Bcl-X_L_/Mcl-1-siRNA treatment. No alteration of Bad or Bik mRNA levels were observed (Figure 4e and f), whereas Hrk was upregulated, however, in a Bak-dependent manner (Figure 4g).

Together, the data suggest that most BH3-only proteins are not induced. Some BH3-only proteins were induced in a Bak-dependent way, suggesting that this induction is downstream of Bak. Therefore expression changes of these molecules very likely do not function as a trigger for apoptosis initiation.

### Individual BH3-only proteins are not required for apoptosis induction upon loss of Bcl-X_L_/Mcl-1

**Figure 5:**
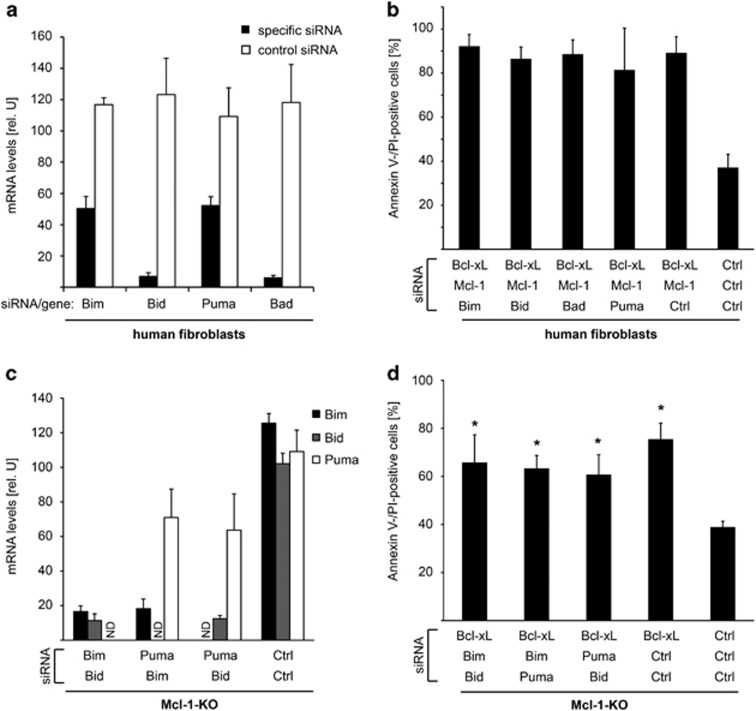
Individual BH3-only proteins are not required for apoptosis induction upon loss of Bcl-X_L_/Mcl-1. (**a**) Primary human fibroblasts were transfected with Bcl-X_L_ and Mcl-1 siRNA together with control or siRNA specific for Bim, Bid, Puma or Bad as indicated. Respective gene expression was analyzed 24 h after transfection. (**b**) Primary human fibroblasts were treated with siRNAs as indicated. Cell death was measured 3 days after transfection. (**c**) MEFs from Mcl-1-deficient mice were treated with two siRNAs as indicated. mRNA levels were measured 24 h after transfection. (**d**) Mcl-1-deficient MEFs were transfected with two BH3-only targeting or control siRNAs as indicated. Forty-eight hours after transfection, cells were transfected with Bcl-X_L_ or control siRNAs and cell death was measured 24 h later. For **a**–**d**, data show means±S.D. of three independent experiments. *P≤0.05; ND, not determined

To examine the relevance of BH3-only proteins more rigorously, we tested whether knock-down of BH3-only proteins affected apoptosis upon targeting of Bcl-X_L_/Mcl-1. We silenced Bim, Bid, Bad or Puma by RNAi in human fibroblasts. siRNA treatment strongly reduced the mRNA levels of the targeted gene (Figure 5a). However, induction of apoptosis was not affected when any of these molecules were targeted along with Bcl-X_L_/Mcl-1 (Figure 5b). The studies were also extended to Mcl-1-deficient MEFs, where two BH3-only proteins, described to directly activate Bax or Bak, were targeted simultaneously. No alteration of apoptosis induction was observed (Figure 5c and d).

These data suggest that individual BH3-only proteins are not required for apoptosis induced by loss of Bcl-X_L_ and Mcl-1, further providing evidence that removal of Bcl-X_L_ and Mcl-1 is sufficient to induce cell death.

### Apoptosis induced by Bcl-X_L_/Mcl-1 inhibition occurs in absence of activator BH3-only proteins

**Figure 6:**
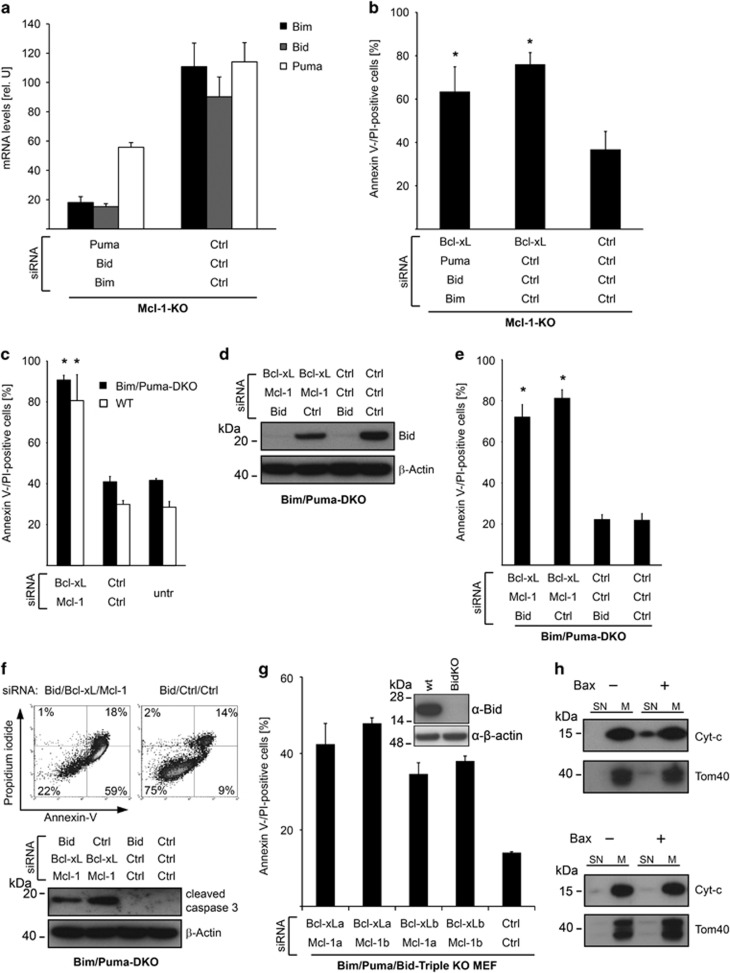
Apoptosis induced by Bcl-X_L_/Mcl-1 inhibition occurs in absence of activator BH3-only proteins. (**a**) Mcl-1-deficient MEFs were treated with control siRNAs or siRNAs targeting Puma, Bid, and Bim simultaneously. mRNA levels were measured 24 h after transfection. Data show means±S.D. of three independent experiments. (**b**) Mcl-1-deficient MEFs were transfected with control siRNAs or siRNAs targeting Puma, Bid and Bim. Forty-eight hour after transfection, cells were transfected with Bcl-X_L_ or control siRNAs and cell death was measured 24 h later. Data show means±S.D. of three independent experiments. (**c**) MEFs from Bim/Puma-double-deficient or WT mice were transfected with Bcl-X_L_ and Mcl-1 or control siRNAs. Cell death was measured 3 days after transfection. (**d**) MEFs from Bim/Puma-double-deficient mice were transfected with control or Bid-specific siRNA for 3 days. Then, cells were seeded and transfected with control, Bcl-X_L_-, Mcl-1- and Bid-specific siRNAs as indicated. Bid was quantified by immunoblotting 3 days after the second transfection. Results are representative of three independent experiments. (**e**) Cell death was measured in Bim/Puma-double-deficient MEFs treated as described in **d**. The mean percentage±S.D. of Annexin V-/propidium iodide-positive cells of three independent experiments is given. (**f**) Upper panel: representative histogram of Bim/Puma-double-deficient MEFs treated as described in **d** indicating cells undergoing apoptosis as demonstrated by an Annexin V-positive/propidium iodide-negative cell population. Lower panel: quantification of caspase-3 activation by immunoblotting. Results are representative of three independent experiments. (**g**) Bid/Bim/Puma-triple-deficient MEFs were transfected with Bcl-X_L_- and Mcl-1-specific siRNAs or control siRNAs. Cell death was assessed after 48 h. For Bcl-X_L_ and Mcl-1 two different siRNA sequences (siRNA a or b) were used in different combinations. Inset shows result of knockout of Bid using CRISPR/CAS9 on Bim/Puma DKO MEF to generate triple KO cells. (**h**) Primary human fibroblasts were treated simultaneously with Bcl-X_L_-, Mcl-1- and Bak-specific siRNAs. Three days after transfection mitochondria were prepared. Mitochondria were treated with recombinant Bax (1000 nM) to stimulate cytochrome *c* (Cyt-*c*) release. Cyt-*c* release was assessed by immunoblotting in supernatant (SN) and mitochondrial (M) fractions. Tom40 (translocase of the outer mitochondrial membrane protein 40) served as a control for mitochondria preparation. (**i**) Mitochondria of Bax/Bak-double-deficient MEFs were treated with recombinant Bax. Cyt-*c* release was measured with 1000 nM Bax. A representative blot of two experiments is shown. *P≤0.05

We next tested whether the combined inhibition of known activator BH3-only proteins may affect cell death. We used Mcl-1-deficient MEFs and successfully silenced Bim, Bid and Puma (Figure 6a). Even simultaneous knock-down of these molecules did not affect apoptosis induction (Figure 6b). We next used MEFs deficient for Bim and Puma. Bcl-X_L_/Mcl-1 targeting induced apoptosis to a similar extent as with WT-MEFs (Figure 6c). Bid was then successfully targeted in these cells by siRNA, providing a second cellular model in which all activator BH3-only proteins are reduced (Figure 6d). Again, cell death upon Bcl-X_L_/Mcl-1 targeting was not altered (Figure 6e). The induction of apoptosis was confirmed by annexin V-positive/propidium staining and by demonstrating the activation of caspase-3 (Figure 6f). To rule out that any activator BH3-only molecule is relevant for apoptosis upon Bcl-X_L_/Mcl-1 loss, we generated MEFs that are triple deficient for Bim, Puma and Bid. For this, CRISPR/Cas9 (clustered regularly interspaced short palindromic repeats/endonuclease) was used to generate Bid-deficient cells on the basis of Bim/Puma-knockout MEFs cells. Triple-knockout cells still strongly underwent apoptosis when Bcl-X_L_/Mcl-1 were targeted using different siRNA sequences (Figure 6g).

To further exclude the involvement of BH3-only proteins we took advantage of a third experimental model and prepared mitochondria from human fibroblasts treated with Bcl-X_L_-, Mcl-1-, and Bak-specific siRNA. These cells survive due to the lack of Bak (Figure 2a). If BH3-only proteins were activated in these cells, they would be localized at the membrane of the isolated mitochondria and would be able to activate exogenously added Bax, leading to release of cytochrome *c*. When active Bim is overexpressed in Bax/Bak double-deficient mitochondria, low levels (about 50 nM) of recombinant Bax are sufficient to cause cytochrome *c* release.^18^ In mitochondria from Bax/Bak double-deficient MEFs that had not been manipulated, high levels (1000 nM) of recombinant Bax caused release of cytochrome *c* (Figure 6h). However, even at this high concentration Bax did not lead to release of cytochrome *c* from mitochondria isolated from human fibroblasts treated with siRNAs targeting Bcl-X_L_, Mcl-1, and Bak (Figure 6i). This indicates that there is no active BH3-only protein at the mitochondria that may have led to Bak activation, when cells have lost Bcl-X_L_ and Mcl-1.

Together these data demonstrate that even the loss of all activator BH3-only proteins does not affect apoptosis induced by inhibition of Bcl-X_L_ and Mcl-1. Further, this occurs in the absence of active BH3-only proteins at mitochondria. This strongly suggests that BH3-only proteins are not involved in apoptosis initiation upon loss of Bcl-X_L_ and Mcl-1 in these cells.

## Discussion

Our results show that the removal of two anti-apoptotic proteins in several non-malignant cell types activates Bak and subsequently causes apoptosis, which appeared not to require BH3-only proteins. This suggests that Bak indeed can auto-activate when this inhibition is removed. These results may provide an explanation why cells express both Bax and Bak despite their apparent biological redundancy.

Arguably, the activation of Bax is better understood than the activation of Bak. Bax can be directly bound by BH3-domains of Bim and Bid.^8^ Bax is normally inactive in the cytosol but very likely can be activated by direct interaction with the BH3-domain of BH3-only proteins. BH3-only proteins (with the exception of Bad) are tail-anchored in the outer mitochondrial membrane.^7, 18^ It seems a likely model that Bax can be activated by BH3-only proteins as it diffuses past the mitochondrial membrane, where it inserts and triggers the release of cytochrome *c*.

It may however not be appropriate to assume the same for Bak. Bak is in the absence of apoptosis already inserted in the OMM. Although Bak can in principle be activated by BH3-only proteins and this may occur through direct binding, the requirements for the activation of Bax *versus* Bak, with their different spatial constraints, are likely different. Further, Bak can bind Bcl-X_L_ and Mcl-1 in principle,^10^ and all three proteins are at mitochondria; it therefore appears likely that there is some binding in a normal, non-apoptotic cell.

Conversely, the indirect activation model is counter-intuitive for Bax, with its primarily cytosolic localization: although the potential binding to anti-apoptotic Bcl-2-proteins for Bax may be suggestive,^11^ Bax is clearly at least predominantly cytosolic. Accordingly, the studies of ‘displacement' have experimentally focused on Bak and have extrapolated the findings to Bax. There is clear evidence that the activation of Bax can involve a measure of displacement, or indirect activation. This however is seen in situations where apoptosis had been initiated but then blocked by anti-apoptotic Bcl-2 proteins. As elaborated on by the ‘unified model' the inhibition of active Bax and Bak is an important function of Bcl-2-like proteins.

Indeed, this way of activation of Bax may be an important difference between tumor cells and non-malignant cells. In many cases tumor development creates the need for alterations that help transformed cells escape defense mechanisms of the host organism such as apoptosis.^19^ We have observed previously that a cell lineage-specific expression profile of anti-apoptotic Bcl-2 proteins is lost in tumor cells, which is associated with increased dependency of tumor cells on these proteins.^15^ Compared with cancer cells, the studies investigating anti-apoptotic Bcl-2 members in non-malignant cell are less comprehensive.

In a study of cancer cells, where a small molecule library was used to identify compounds inhibiting Mcl-1, Bcl-X_L_ was identified as the only marker predicting sensitivity to Mcl-1-inhibiting compounds, and these small molecules induced apoptosis via Bak not Bax.^20^ In the non-transformed cells we used here only the simultaneous removal of Bcl-X_L_ and Mcl-1 but not either on its own (or in combination with any other Bcl-2-like protein) induced apoptosis. We observed this in several non-malignant cell types, indicating a more common survival regulation. This difference between cancer cells and normal cells may be interpreted as an indication of a pre-activated Bcl-2-system in tumor cells. In other cell types this dependency may be different again. Mcl-1 is critically required for the development and maintenance of many cells of the hematopoietic system and it is sufficient to take away Mcl-1 in mature lymphocytes to induce apoptosis.^21^

Together, our study provides evidence that Bak may be activated independently of BH3-only proteins in non-malignant cells. It therefore appears that Bak may auto-activate, and this may be the main mechanism of its activation. Bax and Bak can in many situations have the same activity and replace each other, and there seems no plausible explanation why we have two effector proteins that act exactly the same way. A different mode of activation with Bax requiring activation through BH3-only proteins and Bak auto-activating when the inhibition is removed would appear to provide a reason for the presence of both.

## Materials and Methods

### Reagents and antibodies

Anti–Bcl-2 (Ab-1) antibody was purchased from Merck Biosciences (Darmstadt, Germany). Anti-Bcl-X_L_, anti-Bcl-w, anti-Bid, anti-Bim (C34C5), anti-Bak, anti-caspase-3, anti-caspase-8 (1C12), anti-caspase-9 and HRP-conjugated secondary antibodies were obtained from New England Biolabs (Berlin, Germany). Anti-Mcl-1 (22) antibody was from BD Bioscience (Heidelberg, Germany), anti-Bax (2D2) was from Santa Cruz Biotechnology (Heidelberg, Germany) and anti-cytochrome *c* (clone 7H8.2C12) was purchased from BD Biosciences (Heidelberg, Germany). Anti-*β*-actin (AC-15) and anti-*α*-tubulin (DM1A) antibodies were obtained from Sigma-Aldrich (Munich, Germany). Bax activation was measured via flow cytometry by staining with an antibody specific for the conformation of activated Bax (Clone 3; BD Pharmingen, Heidelberg, Germany). PCR primers and siRNAs were purchased from Eurofins MWG Operon (Ebersberg, Germany).

### siRNAs

siRNAs were designed as described previously.^22^ Sequences of specific siRNAs were A1 (human): GGAAGAAUUGUAACCAUAU, Bad (human): GGATGAGTGACGAGTTTGT, Bak (human): GCTTCGTGGTCGACTTCAT, Bak (mouse): ACACAGAGTTCCAGAATTT, Bax (human): GGGTTGTCGCCCTTTTCTA, Bax (mouse): GATGAACTGGACAGCAATA, Bcl-2 (human): GGGAGAUAGUGAUGAAGUA, Bcl-w (human): GGCAGACUUUGUAGGUUAU, Bcl-X_L_ (human): GGAACUCUAUGGGAACAAU, Bcl-X_L_ (mouse): GCGTGGAAAGCGTAGACAA, Bid (human): CTTGCTCCGTGATGTCTTT, Bid (mouse): CGACTGTCAACTTTATTAA, Bim (human): GCAACCTTCTGATGTAAGT, Bim (mouse): GGAGACGAGTTCAACGAAA, Mcl-1 (human): GGCAGUCGCUGGAGAUUAU, Mcl-1 (mouse): GGGACTGGCTTGTCAAACA, Puma (human): CCGAGATGGAGCCCAATTA, and Puma (mouse): AUGCACUGCUGUAGAUAUA. Depicted is the 19 nt portion corresponding to the sense strand of the targeted mRNA. The sequence of the non-silencing control siRNA (Ctrl) was GCGCAUUCCAGCUUACGUA.

### Generation of Bim, Puma, Bid triple knockout MEF using CRISPR/Cas9

Bid was specifically targeted in Bim/Puma double-deficient MEF by using a two vector based CRISPR/CAS9 system kindly provided by Dr. Veit Hornung (Institute of Molecular Medicine, University Hospital Bonn, Germany) by transient transfection and subsequent selection. Efficacy was tested using antibody against Bid.

### Cell culture

Primary human fibroblasts and keratinocytes were isolated from neonatal human foreskins. Cultivation of cells was described previously.^23^ Human microvascular endothelial cells and immortalized murine embryonal fibroblasts (MEFs) were cultivated in DMEM (Dulbecco's modified Eagle's medium) medium supplemented with 10% FCS (fetal calf serum).

### Transfection procedures

siRNA transfections were performed in 6-well dishes in a volume of 1 ml utilizing Lipofectamine RNAiMAX (Invitrogen, Darmstadt, Germany) according to the manufacturer's protocol. For inhibition of a single gene, 20 nM siRNA together with 1.25 *μ*l Lipofectamine RNAiMAX was used. For dual or triple inhibition experiments, each specific siRNA was used in a concentration of 20 nM. The amount of control siRNA was adjusted to the total amount of specific siRNAs used in the experiment as indicated in the figures. In dual inhibition experiments 40 nM specific siRNAs or 40 nM control siRNA, in triple inhibition experiments 60 nM siRNAs or 60 nM control siRNA was used together with 2 *μ*l Lipofectamine RNAiMAX. Plasmides were transfected at 1 *μ*g/ml with 3 *μ*l FuGENE 6 (Roche) according to the manufacturer's protocol.

### Quantification of viable cells

Viable cells with intact metabolism were identified by their ability to reduce cell-permeable resazurin to fluorescent resorufin. Medium was replaced with 750 μl of culture medium and 150 *μ*l of CellTiter-Blue reagent (CellTiter-Blue Cell Viability Assay; Promega, Mannheim, Germany). After 1 h incubation at 37 °C, fluorescence was measured.

### Quantification of apoptotic cells and cell death

Adherent and supernatant cells were analyzed by staining with FITC-labeled Annexin V (Roche, Mannheim, Germany) and propidium iodide (Sigma-Aldrich) as described previously.^24^ Cells were analyzed by fluorescence-activated cell sorting analysis using CellQuest software (BD Bioscience).

### RNA extraction and quantification

Total RNA was extracted from cells using QIAzol Lysis Reagent (Qiagen, Hilden, Germany) as described by the manufacturer and analyzed by quantitative RT-PCR. Reverse transcription and quantification procedures using the LightCycler TaqMan Master Kit (Roche) together with the Universal Probe Library system (Roche) were described previously.^24^ Relative gene expression was expressed as a ratio of the expression level of the gene of interest to that of hypoxanthine phosphoribosyltransferase RNA determined in the same sample.

### Protein preparation and immunoblot analysis

Adherent and supernatant cells were lysed in buffer containing 50 mM Tris, pH 7.4, 0.25 M NaCl, 1 mM EDTA, 0.1% Triton X-100, phosphatase inhibitors (PhosSTOP, Roche) and protease inhibitors (Complete, Mini, EDTA-free; Roche). Gel electrophoresis, blotting and protein detection was carried out using the Xcell *SureLock* Mini-Cell apparatus (Invitrogen) as described previously.^24^ Protein levels of *β*-actin or *α*-tubulin were analyzed as a control for constant loading and transfer.

### Isolation and treatment of mitochondria

Mitochondria were isolated with a cell homogenizer (Isobiotec, Germany) pre-cooled on ice together with a 10 mm tungsten carbide ball inserted. The homogenizer was equilibrated with isolation buffer (PBS supplemented with protease inhibitor cocktail for mammalian cell (Sigma-Aldrich)). A total of 5 × 10^6^ cells were resuspended in 1 ml of isolation buffer and cell membranes were ruptured by passing the cell suspension three times through the system. The homogenate was recovered by rinsing once with 1 ml of isolation buffer and centrifuged (800 × *g*, 5 min at 4 °C) to remove cell debris and nuclei. The supernatant was centrifuged at 9000 × *g* (10 min at 4 °C) and the mitochondria-containing pellet was resuspended in 50 *μ*l of isolation buffer. A total of 30 *μ*g of the mitochondrial sample were centrifuged (9000 × *g*, 10 min at 4 °C) and resuspended in 20 *μ*l of KCl buffer (10 mM HEPES pH 7.4, 125 mM KCl, 4 mM MgCl_2_, 5 mM KH_2_PO_4_ and 0.5 mM EGTA), supplemented with Bax as indicated in respective experimental conditions. Mitochondria were incubated for 30 min at 30 °C and mitochondria were pelleted at 9000 × *g* for 10 min at 4 °C. Supernatant was collected and pelleted mitochondria were washed in 150 *μ*l KCl buffer and resuspended in 20 *μ*l of KCl buffer. Supernatant and mitochondrial fractions were analyzed by immunoblotting.

### Statistics

For statistical analysis, two-tailed Student's *t*-test was used to assess the significance of mean differences. Differences were considered significant at a *P*-value of 0.05 or less (indicated in the Figures (n.s., not significant; *P*>0.05)).

## Abbreviations

Bax: Bcl-2-associated X protein
Bak: Bcl-2 homologous antagonist/killer
Bcl-X_L_: B-cell lymphoma-extra large
Bcl-2: B-cell lymphoma 2
Bcl-w: Bcl-2-like protein 2
Mcl-1: myeloid cell leukemia sequence 1
A1: Bcl-2-related protein A1
Bim: Bcl-2-interacting mediator of cell death
Bid: BH3-interacting domain death agonist
Puma: p53-upregulated modulator of apoptosis
Noxa: Phorbol-12-myristate-13-acetate-induced protein 1
Bad: Bcl-2-associated death promoter
Bmf: Bcl-2-modifying factor
Bik: Bcl-2-interacting killer
Hrk: activator of apoptosis hara-kiri
CRISPR: clustered regularly interspaced short palindromic repeats
Cas9: endonuclease
